# Clinical efficacy of needle aspiration combined with closed reduction and internal fixation within 24–72 h in the treatment of pediatric femoral neck fracture

**DOI:** 10.3389/fped.2026.1768628

**Published:** 2026-03-20

**Authors:** Jingjing Zuo, Xiaodong Yang, Jun Jiang, Li Zou, Lijun Liu, Xueyang Tang, Lei Yang

**Affiliations:** 1 Rehabilitation Medicine Centre, West China Hospital, Sichuan University, Chengdu, Sichuan, China; 2 Department of Pediatric Surgery, West China Hospital, Sichuan University, Chengdu, Sichuan, China

**Keywords:** avascular necrosis, capsular decompression, closed reduction and external fixation, needle aspiration, pediatric femoral neck fracture

## Abstract

**Background:**

Femoral neck fracture is a less common injury in childhood but carry a high risk of avascular necrosis (AVN) of the femoral head. This study aimed to assess the clinical efficacy of needle aspiration combined with closed reduction and internal fixation (CRIF) performed within 24–72 h of injury for the treatment of pediatric femoral neck fractures.

**Methods:**

This retrospective study enrolled 26 children with femoral neck fractures who underwent cannulated screw fixation using CRIF at our department between January 2010 and January 2014. Based on whether needle aspiration was performed after fixation, patients were stratified into two groups: the aspiration group (AG, *n* = 11) and the non-aspiration group (NG, *n* = 15). All patients were followed for a minimum of 5 years. The primary outcomes were the effects of needle aspiration and the complication of AVN. Secondary outcomes included functional outcomes assessed using Ratliff’s criteria, as well as the incidence of coxa vara and non-union. According to Ratliff’s criteria, a good result was defined as a “satisfactory outcome,” while fair and poor results were categorized as “unsatisfactory outcomes.”

**Results:**

A total of 26 patients (26 hips) were evaluated, including 14 girls and 12 boys, with a mean age of 9.6 ± 2.5 years (range, 5–14 years). No significant differences were observed between these two groups in baseline characteristics, including gender, age, affected side, type of fracture, displacement, and time to reduction. The rate of satisfactory outcomes was 81.8% in the AG group and 66.7% in the NG group (OR = 2.25, CI, 0.35–14.61, *p* = 0.658). AVN occurred in six cases overall, with two cases in the AG group and four in the NG group (OR=1.63, CI, 0.24–11.08, *p* = 1.000). Based on the Ratliff classification system for AVN, four cases were classified as grade I, one as grade II, and one as grade III. Logistic regression analysis identified reduction quality as an independent risk factor for the development of AVN (OR = 12.06, CI, 1.01–143.60, *p* = 0.049). There was no significant difference in the incidence of other complications between these two groups.

**Conclusions:**

For children under 14 years of age with type II and III femoral neck fractures, needle aspiration combined with CRIF performed within 24–72 h of injury did not yield a statistically significant reduction in the risk of AVN compared with primary CRIF alone. Reduction quality was confirmed as an independent risk factor for postoperative AVN in this pediatric population.

## Introduction

Femoral neck fractures are relatively uncommon in children, accounting for less than 1% of all pediatric fractures and 7.0%–12.8% of all pediatric femoral fractures (1, 2). However, a high risk of complications has been reported in the literature, including avascular necrosis (AVN) of the femoral head, coxa vara, fracture non-union, premature physeal arrest, and others (3). Among these, AVN is the most common complication, often leading to hip arthritis and unsatisfactory outcomes (4). The literature has reported various attempts to identify risk factors for the development of AVN, such as age, fracture type, time to fixation, reduction quality, and capsular decompression (4–6). In clinical practice, performing early or immediate capsular decompression or needle aspiration may be challenging for various reasons; however, a delayed procedure performed in conjunction with reduction is more feasible. The role of needle aspiration in preventing AVN remains unclear, and few studies have focused on this issue (6, 7).

Therefore, this retrospective study aimed to evaluate the clinical efficacy of needle aspiration combined with closed reduction and internal fixation (CRIF) performed within 24–72 h of injury for the treatment of pediatric femoral neck fractures.

## Materials and methods

This study was reviewed and approved by the Human and Ethics Committee for Medical Research at Sichuan University in accordance with the Declaration of Helsinki (Committee reference number: 1085, Chengdu, Sichuan, 2018). Written informed consent regarding publishing their data and photographs was obtained from the parents of all pediatric participants. The inclusion criteria were as follows: patients younger than 14 years, fresh fractures treated surgically within 3 days of injury, treatment with CRIF, and a minimum follow-up of 5 years with complete data. The exclusion criteria included treatment with a conservative protocol or open reduction, pathological fractures, delayed reduction beyond 3 days, and type IV fractures according to the Delbet classification (8). A total of 41 potential patients were initially screened between January 2010 and January 2014. Following strict evaluation, 26 patients met the eligibility criteria, while 15 patients were excluded (including four cases with delayed treatment beyond 72 h, six who underwent open procedures, two with pathological fractures, and three with type IV fractures). Based on whether needle aspiration was performed after fixation, the eligible patients were divided into two groups: the aspiration group (AG, *n* = 11) and the non-aspiration group (NG, *n* = 15). All 26 enrolled patients underwent regular follow-up after treatment.

Surgery was performed by two senior pediatric orthopedic surgeons well-trained in this technique under general anesthesia. Fewer than five attempts at closed reduction with manipulation were allowed. Otherwise, open reduction was considered. Two cannulated screws (IDEAL MEDICAL, China) were implanted to maintain fracture reduction. To differentiate it from early or immediate aspiration after injury, we defined aspiration performed with a reduction procedure between 24 and 72 h after injury as delayed aspiration. For the AG group, a 20-G lumbar puncture needle was used to perform percutaneous joint aspiration. Intraoperative pressure measurement was not performed in this study. All patients in the aspiration group achieved successful fluid aspiration, which appeared as light red to dark red bloody fluid. Ultrasound was used to confirm both the presence of fluid and the success of aspiration. Postoperatively, a hip spica was applied to all patients for approximately 6–8weeks. Partial weight-bearing was initiated after removal of the spica, and full weight-bearing was permitted 12 weeks after surgery. Internal fixation devices were usually removed 12–24 months after operation in the presence of satisfactory fracture healing on plain radiographs. This group of patients was followed for at least 5 years, with a mean follow-up of 6.8 ± 1.6 years (range, 5–8 years). Follow-up visits were scheduled every 2 weeks during the first month, monthly during the second and third months, every 3 months during the first year, and annually from the second year onward.

Baseline data were collected from hospital records, including gender, age, affected side, fracture type based on the Delbet grading system, displacement, and time to reduction. Two independent pediatric orthopedists conducted the radiological and clinical evaluation in a blinded and independent manner, with statistical analysis verifying high inter-rater agreement. The primary outcomes were the effects of needle aspiration and the complication of AVN. The initial diagnostic criteria for AVN were based on plain radiographic findings. Coxa vara was measured using full-length lower extremity radiographs and was defined as a neck–shaft angle of less than 110°. Secondary outcomes included the functional outcomes assessed using Ratliff's criteria (9), as well as the incidence of coxa vara and non-union. Reduction quality was categorized into three types based on Song's classification system (10): (1) anatomical reduction with no displacement or angular deformity; (2) acceptable reduction with a displacement of <2 mm or angular deformity within 20° of the normal neck–shaft angle; and (3) unacceptable reduction with a displacement of >2 mm or angular deformity >20° of the normal neck–shaft angle. According to Ratliff’s criteria, a good result was defined as a “satisfactory outcome,” while the fair and poor results were categorized as “unsatisfactory outcomes.”

SPSS 29 was used for data analysis. Continuous data were reported using mean ± standard deviation and range. Categorical data were reported as numbers and percentages. Comparisons of continuous data between these two groups were performed using an independent-samples *t*-test, and the Shapiro–Wilk test was used to test the normality of data distribution. For comparisons of enumeration data, Fisher's exact test was used. Risk factors were evaluated using a logistic regression model, and odds ratios (ORs) with 95% confidence intervals (CIs) were also calculated. A *p*-value <0.05 was considered significant.

## Results

**Figure 1:**
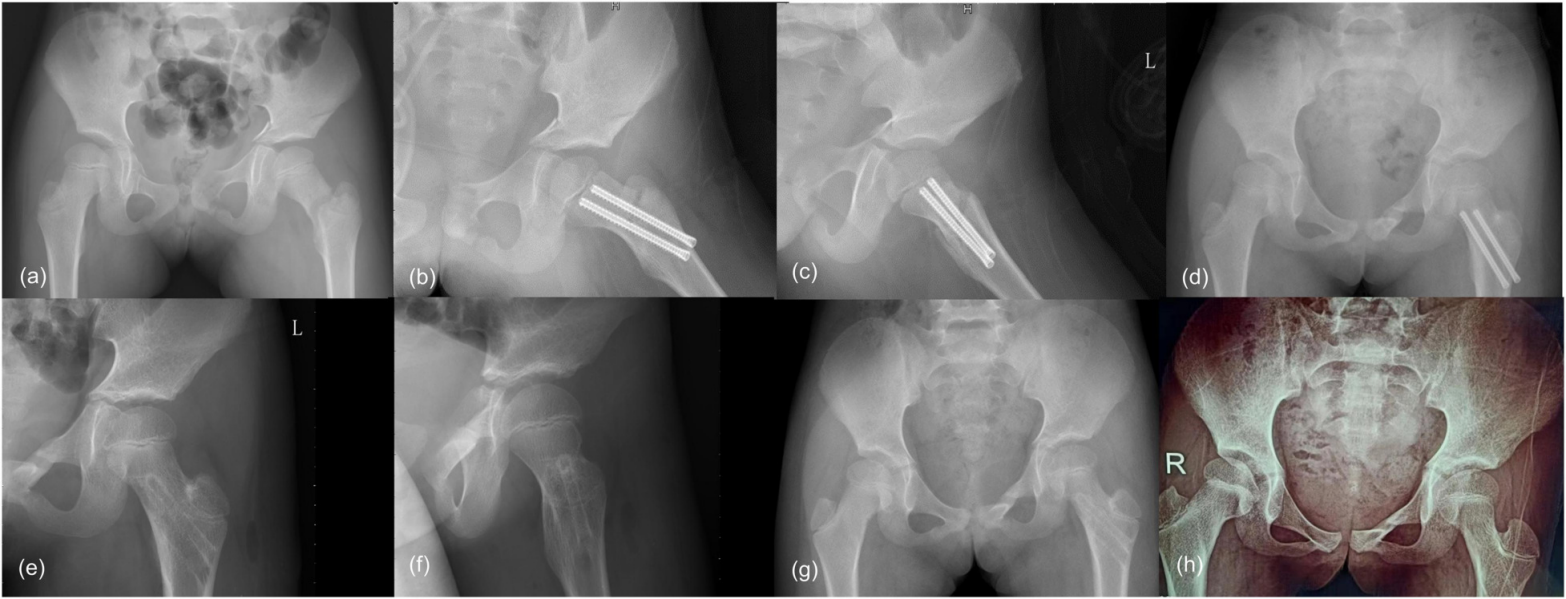
Radiographic outcomes of a 7-year-old girl with Delbet type III femoral neck fracture on the left side. **(a)** Preoperative pelvic radiograph. **(b** and **c)** Postoperative radiographs of the femoral neck on the first day after the operation. **(d)** Postoperative pelvic radiograph 1 year after the operation. **(e** and **f)** Radiograph of the femoral neck on the first day after hardware removal. **(g)** Radiograph of the pelvic 2 years after the initial surgery. **(h)** Radiograph of the pelvic 4 years after the initial surgery.

A total of 26 patients (26 hips) were evaluated, including 17 left-sided and nine right-sided fractures. The cohort comprised 14 girls (53.8%) and 12 boys (46.2%), with a mean age of 9.6 ± 2.5 years (range, 5–14 years). According to the Delbet classification, 17 cases (65.4%) were type II and nine cases (34.6%) were type III (Figure 1). No Delbet type I fractures were included in our study because the only two identified cases of type I fractures required open reduction procedures and were therefore excluded. A total of 21 cases (80.8%) presented with displaced fractures, including 8 cases in the AG group and 13 cases in the NG group. The average time from injury to fracture reduction was 38.5 ± 20.2 h (range, 24–72 h). All these baseline data showed no significant difference between the AG and NG groups. Detailed data are presented in Table 1.

**Table 1:** Demographic data and outcomes of the patients with femoral neck fractures.

Characteristics	AG	NG	*P*-value
Gender			0.692
Female	5	9	
Male	6	6	
Age, years	9.7 ± 2.7	9.5 ± 2.5	0.801
Affected side (left/right)	7/4	10/5	1.000
Type of fracture			0.217
Delbet type II	9	8	
Delbet type III	2	7	
Displaced fracture	8	13	0.620
Time to reduction	39.7 ± 23.1	37.6 ± 18.6	0.797
Reduction quality			1.000
Anatomic	8	10	
Acceptable	3	5	
Transphyseal screw	2	4	1.000
Outcome			0.658
Satisfactory	9	10	
Unsatisfactory	2	5	
AVN	2	4	1.000
Coxa vara	1	3	0.614

AG, aspiration group; NG, non-aspiration group; AVN, avascular necrosis.

Anatomical reduction was achieved in 18 cases (69.2%), while acceptable reduction was achieved in eight cases (30.8%). Statistical analysis revealed that reduction quality was a significant risk factor for AVN (OR = 12.06, CI, 1.01–143.60, *p* = 0.049). Transphyseal screw placement was noted in six cases (23.1%), and this factor was not significantly associated with the risk of AVN (OR = 2.00, CI, 0.27–15.08, *p* = 0.596). Detailed data are presented in Table 2.

**Table 2:** Multivariate analysis for risk factors of avascular necrosis of the femoral head.

Characteristics	Risk factors	Odds ratio	95% CI	*P*
Avascular necrosis of femoral head	Displacement	0.33	0.01–7.91	0.490
	Fracture type	1.06	0.10 −11.02	0.963
	Reduction quality	12.06	1.01–143.60	**0.049**

CI, confidence interval. Bold values indicate statistically significant results (*p* < 0.05).

According to Ratliff's criteria, 19 cases (73.1%) had a good outcome, 6 (23.1%) cases had a fair outcome, and 1 case (3.8%) had a poor outcome. The rate of satisfactory outcomes was 81.8% in the AG group and 66.7% in the NG group (OR = 2.25, CI, 0.35–14.61, *p* = 0.658). During follow-up, AVN developed in six cases, including two cases in the AG group and four cases in the NG group (OR = 1.63, CI, 0.24–11.08, *p* = 1.000). The mean time to AVN detection was 11.0 ± 4.1 months (range, 6–18 months). Based on the Ratliff classification system for AVN (9), four cases were classified as type III, one as type II, and one as type I. Coxa vara occurred in one case in the AG group and three cases in the NG group (*p* = 0.614). No cases of bone non-union were observed.

## Discussion

Femoral neck fractures are rare injuries in children but are associated with a high risk of complications (6, 11). AVN is one of the most common complications and can occur following both conservative and surgical treatment (12, 13). Bali et al. (14) believed that conservative treatment carries a high risk of complications (60.0%) and failure to maintain reduction (61.5%). Thus, surgical treatment with internal fixation should be considered whenever feasible. The selection of open or closed reduction remains controversial and usually depends on the type of fracture and the ability of the surgeon (6). Some authors have reported better outcomes with open reduction compared to closed reduction, primarily due to more satisfactory reduction and the ability to perform capsular decompression (10, 15). Stone et al. (16) reported a lower rate of AVN after open reduction and fixation (0%) compared with CRIF (50%) and better outcomes in reduction quality and bone union. However, there remains concern about disrupting the vascular supply during open reduction, which may increase the risk of AVN (17, 18). In a recent study by Wu et al. (13) on delayed reduction in pediatric femoral neck fracture, they reported better efficacy and a lower risk of AVN with closed reduction compared to open reduction. AlKhatib et al. (19) reported no significant association between the method of reduction and the risk of AVN based on the current evidence. In our opinion, CRIF represents an effective and less invasive option that could be considered before attempting open reduction. However, the closed procedure should be performed gently, and multiple attempts should be avoided.

Time to reduction has been considered a predictor of AVN, and many authors have reported better outcomes with early reduction compared to delayed reduction (20). In a systematic review by Yeranosian et al. (21), a delay in treatment beyond 24 h was associated with a higher incidence of AVN. Papakostidis et al. even suggested that the reduction procedure should be completed within 12 h (22). In contrast, there were still differing views on the effect of early vs. delay treatment. In a recent systematic review by AlKhatib et al. (19), they reported that the current evidence does not indicate an association between time to treatment and the risk of AVN. Koroglu et al. (23) also found no significant correlation between the development of AVN and the timing of surgery. Although controversy remains, most studies advocate early intervention when clinical conditions are feasible (7, 20).

In our practice, an early reduction was also recommended for patients with stable vital signs and conditions permitted. As a regional trauma center, we received many patients transferred from local hospitals, and most emergency surgeries in our hospital can be prepared and performed within 12 h. However, in our study, the average time to reduction was 38.5 h, which was mainly due to the long time spent on transfer from the initial hospital. By the time patients arrived at our emergency center, 12 h or more had often already passed, making it difficult to achieve early reduction. Similar delays in reduction have also been found in other literature works and, in some cases, may be even longer (13, 22).

The role of decompression in reducing the incidence of AVN remains unclear due to the paucity of relevant studies and the limited evidence available (6, 24). To achieve decompression, open capsulotomy or joint aspiration can be used. Bukva et al. (25) reported that hip decompression has a positive effect on reducing the risk of AVN and that open capsulotomy decompression was as effective as needle aspiration decompression. Some authors even believed that open surgery could achieve better outcomes, mainly because it allows for good reduction and open capsular decompression during the procedure; however, there is a lack of direct evidence (2, 26). Some previous studies have not shown any benefit of decompression in decreasing the risk of AVN (21). In a long-term follow-up study by Varshney et al. (27), capsular decompression had no effect on the outcomes of delayed reduction. A similar result was also supported by Spence (11) in a study examining risk factors for AVN. In our study, needle aspiration performed within 24–72 h of injury showed no significant reduction in the risk of AVN after CRIF.

Despite numerous identified risk factors for AVN of the femoral head, fracture reduction quality remains one of the most widely discussed factors, with controversy conclusions in the literature (23, 28). In this study, reduction quality was confirmed as an independent risk factor for postoperative AVN in this pediatric population. However, the small sample size resulted in a wide 95% confidence interval, limiting the precision of our estimate and the ability to draw definitive conclusions. Based on our clinical practice, achieving high-quality anatomic reduction remains clinically essential when feasible.

Since this was a retrospective study with a small sample size, we acknowledge the limited statistical power, which will be further elaborated in the limitations section. Other limitations of our study include the absence of intraoperative pressure measurements and aspiration volumes. We cannot equate percutaneous needle aspiration with capsular decompression. In addition, aspiration in our study was performed during the reduction procedure, which delayed the time from injury to aspiration and was not an early intervention. Therefore, further research focusing on early aspiration or capsular decompression is needed.

## Conclusions

For children under 14 years of age with type II and III femoral neck fractures, needle aspiration combined with CRIF performed within 24–72 h of injury did not yield a statistically significant reduction in the risk of AVN compared with primary CRIF alone. However, reduction quality was confirmed as an independent risk factor for postoperative AVN in this pediatric population.

## Data Availability

The raw data supporting the conclusions of this article will be made available by the authors, without undue reservation.
